# Predictive value of neutrophil to lymphocyte ratio for the clinical outcomes of acquired immune deficiency syndrome: a systematic review and meta-analysis

**DOI:** 10.3389/fmed.2025.1503614

**Published:** 2025-02-05

**Authors:** Fuyu Guo, Jiamei Chen, Hengkai Zhang

**Affiliations:** ^1^The Second Clinical Medical College of Jilin University, Changchun, China; ^2^School of Public Health, Jilin University, Changchun, China; ^3^China-Japan Union Hospital of Jilin University, Changchun, China

**Keywords:** HIV, AIDS, NLR, prognosis, meta-analysis, mortality, PFS

## Abstract

**Objective:**

This study aimed to explore the predictive value of the neutrophil-to-lymphocyte ratio (NLR) for outcomes in Acquired Immune Deficiency Syndrome (AIDS) patients.

**Methods:**

PubMed, Embase, Cochrane, and Web of Science were conducted to search literature up to May 2024 and cohort and case–control studies were included. The primary outcomes were mortality and progression-free survival (PFS). Pooled odds ratios (ORs) or hazard ratios (HRs) with 95% confidence intervals (CIs) were calculated using a random-effects model. We conducted sensitivity analyses to assess result stability, reliability, and subgroup analyses to identify sources of heterogeneity using Review Manager 5.4.1. Egger’s tests were performed with Stata 15.1, and funnel plots were generated using Review Manager 5.4.1. Microsoft Excel was used for the initial data summarization.

**Results:**

Fourteen studies involving 30,752 AIDS patients were included. The pooled data showed higher NLR significantly associated with increased mortality (OR: 1.85, 95% CI: 1.43–2.41, *p* < 0.00001) and shorter progression-free survival (PFS) (HR: 2.46, 95% CI: 1.32–4.59, *p* = 0.005). Subgroup analyses revealed that NLR’s predictive value was greater in studies with post-ART measurements. Sensitivity analyses show stable and reliable results. Egger’s test and funnel plot analysis revealed no significant publication bias.

**Conclusion:**

NLR is a key prognostic biomarker for predicting mortality and progression-free survival (PFS) in AIDS patients. Incorporating NLR into predictive models may improve prognostic assessments and guide clinical decision-making.

**Systematic Review Registration:**

PROSPERO (CRD42024532918: https://www.crd.york.ac.uk/PROSPERO).

## 1 Introduction

Since its identification in the early 1980s, the human immunodeficiency virus (HIV) has become a major global health issue, affecting millions worldwide (1). In 2022, an estimated 39 million people (33.1 million to 45.7 million) were living with HIV around the world. Consequently, antiretroviral therapy (ART) is the standard treatment for HIV-positive individuals. ART typically involves administering at least three antiretroviral (ARV) drugs to maximize HIV suppression and prevent the progression of related diseases. Research has shown that effective ART significantly reduces mortality rates (2).

Age, IL-6, D-dimer, CRP, tissue fibrosis (HA), and fibrinogen have been strongly associated with all-cause mortality in AIDS patients. Moreover, interruption of antiretroviral therapy may increase this risk (3–7). Lymphopenia also serves as an independent risk factor for poor prognosis in AIDS patients. The neutrophil-to-lymphocyte ratio (NLR), a novel inflammatory marker, represents the ratio of absolute neutrophil to lymphocyte values in peripheral blood. Recently, NLR has drawn researchers’ attention due to its correlation with the incidence of many chronic diseases (8, 9), mortality from advanced diseases, and poor prognosis in various malignancies, such as lung, gastric, colorectal, pancreatic, breast, and ovarian cancers (10, 11). Additionally, the simplicity and low cost of measuring NLR contribute to its widespread use. NLR has been found to correlate with all-cause mortality in AIDS patients. For instance, Pinato et al. included 9 patients with HIV-associated HCC secondary to hepatitis C (69%) and hepatitis B virus infection (32%). The median survival was 22 months, and an elevated NLR was found to indicate a poorer prognosis (HR: 5.7, 95% CI: 1.5–21.3) (12). Similarly, Deng et al. included 57 patients with colorectal cancer combined with HIV who underwent surgery at the Shanghai Public Health Clinical Center from January 2015 to December 2021. After a follow-up of 3–86 months, a low NLR was an independent predictor of better mortality and PFS (mortality: HR 0.094, 95% CI:0.02–0.45, *p* = 0.003; PFS: HR 0.265, 95% CI: 0.088–0.8, *p* = 0.019) (13).

The correlation between NLR and poor prognosis in HIV-positive patients with solid tumors has been demonstrated. However, this relationship lacks validation from an evidence-based medicine perspective. This study aimed to investigate the association between NLR and prognosis in HIV-infected patients.

## 2 Materials and methods

### 2.1 Literature search strategy

This study followed the PRISMA 2020 guidelines (14) and was registered on PROSPERO (CRD42024532918). Relevant literature was searched in PubMed, Embase, Cochrane, and Web of Science databases from inception to May 2024. The search strategy utilized MeSH terms, including “Neutrophil,” “LE Cells,” “Lymphocyte,” “Lymphoid Cells,” “Acquired Immune Deficiency Syndrome,” “AIDS,” “HIV,” and “NLR.” The detailed search strategy is provided in Supplementary Table S1.

### 2.2 Inclusion and exclusion criteria

Inclusion criteria: (1) P: Adult HIV-positive patients; (2) E: Intervention group with high NLR; (3) C: Intervention group with low NLR; (4) O: At least one outcome, including end of follow-up, death, or occurrence of a cardiovascular event; (5) S: Case–control or cohort studies. Exclusion criteria: (1) animal experiments, conference abstracts, case reports, systematic reviews, or letters; (2) studies with non-extractable data.

### 2.3 Data extraction and quality assessment

From each included article, we extracted the following information: first author, year of publication, study area, study type, demographic characteristics, timing of data testing, years of follow-up, NLR cut-off values, laboratory data (WBC, CD4, HIV viral load, neutrophil count, lymphocyte count, albumin), and OR or HR with 95% CI for mortality and PFS. Data extraction was performed independently by the first and second authors, with discrepancies resolved through consensus. Table 1 presents the included studies’ characteristics. The Newcastle-Ottawa Quality Assessment Scale (NOS) was used to evaluate the included studies based on subject selection, comparability, outcome, and exposure factors, with a maximum score of 9 (15). Studies scoring 7 or above were considered high quality (15). See Supplementary Table S2 for details.

**Table 1:** Basic characteristics of the included literature.

Author	Study period	Region	Study type	Population	Time of test	Follow-up(year)	No. of patients	Male/Female	Mean/Median Age	CD4(cells/mm^3^)	CD4/CD8	NLR cut-off
Quiros-Roldan et al.	2000–2013	Italy	Retrospective	Mean age of first diagnosed HCC hiv-infected individuals 38.1 years old	Baseline/post-treatment	8.3	40	36/4	38.1	332.1	NA	2.9
Raffetti et al.	2000–2012	NA	Retrospective	HIV-infected patients with a mean age of 38.4 years in the Italian MASTER cohort	Baseline	3.9	8,230	6013/2217	38.4	395.5	0.4	1.1
Vaughan et al.	2019–2022	South African	Prospective	Mean age of patients diagnosed with DLBCL 42 years old	Baseline/post-treatment	1.58	62	34/28	41	143	NA	6
Postorino et al.	2000–2015	NA	Retrospective	Mean age of HIV-infected patients aged 42.7 years treated with atazanavir but not ritonavir in the Italian MASTER cohort	Post-treatment	2.41	436	268/168	42.7	486.4	NA	NA
Raffetti-b et al.	1998–2012	NA	Retrospective	Mean age 43.2 years of patients with non-Hodgkin’s lymphoma combined with hiv infectionin the Italian MASTER cohort	Baseline/posttreatment	2	215	172/43	43.2	275.9	0.46	3
Raffetti-a et al.	1998–2012	NA	Retrospective	HIV-infected patients with a mean age of 46.2 years in the Italian MASTER cohort	Baseline/posttreatment	3.2	573	437/136	46.2	380.5	0.56	5
Pinato et al.	2001–2014	Italy ; UK	Prospective	Patients aged 36–71 years diagnosed with HCC combined with HIV	Baseline	2.41	59	54/5	52	424	NA	5
Deng et al.	NA	China	Retrospective	HIV-infected persons with a diagnosis of Colorectal Cancer at a median age of 60 years	Baseline/posttreatment	7.16	57	49/8	60	305.3	0.5	2.8
Sanchez et al.	2003–2018	Colombia	Retrospective	HIV-infected patients diagnosed with non-Hodgkin’s lymphoma	Baseline/posttreatment	2	31	NA	NA	NA	NA	4.35
Hanberg et al.	2012–2016	NA	Prospective	Veterans infected with HIV with a mean age of 52.9 years	posttreatment	5	15,594	15,197/397	NA	NA	NA	NA
Miyahara et al.	2002–2015	Thailand	Prospective	HIV-infected individuals with a negative TB screen who are on average 35 years of age	Baseline/posttreatment	0.86	1,118	NA	35	NA	NA	2
Quiros-Roldan et al.	2000–2012	Italy	Retrospective	HIV-infected persons with previously undiagnosed cardiovascular disease with a mean age of 38.1 years	Baseline/posttreatment	8.3	3,766	2685/1081	38.1	441.9	0.49	1.2
Baluku et al.	2008–2018	Uganda	Retrospective	HIV-infected persons with or without lung cancer with a median age of 46 years	Baseline	NA	115	32/83	46	NA	NA	2.44
Ou-Yang et al.	2017–2021	China	Retrospective	HIV-infected persons with or without hypertension with a median age of 51 years	Posttreatment	NA	456	292/164	51	397.1	0.7	NA

Baseline, Not on antiretroviral therapy at the time of testing; post treatment, Already on antiretroviral therapy at the time of testing; NA” indicated that Comorbidities were no mentioned in the study.

### 2.4 Statistical method

The meta-analysis was conducted using Review Manager 5.4.1. OR or HR with 95% confidence intervals (CIs) were used for data synthesis. Heterogeneity was assessed with the chi-squared (*χ*^2^) test (Cochran’s Q) and inconsistency index (*I*^2^). A *χ*^2^ *p* value <0.1 or *I*^2^ > 50% indicated significant heterogeneity. A random-effects model calculated the pooled OR or HR for each outcome. Subgroup analyses were performed for outcomes with at least five studies to assess potential confounders, where data were sufficient. Sensitivity analysis was performed to evaluate the impact of each included study on the pooled OR or HR for outcomes with at least three studies. Funnel plots in Review Manager 5.4.1 and Egger’s regression tests in Stata 15.1 (Stata Corp, College Station, Texas, United States) were used to evaluate publication bias. A *p* value <0.05 indicated statistically significant publication bias.

## 3 Results

### 3.1 Identification of relevant studies

**Figure 1:**
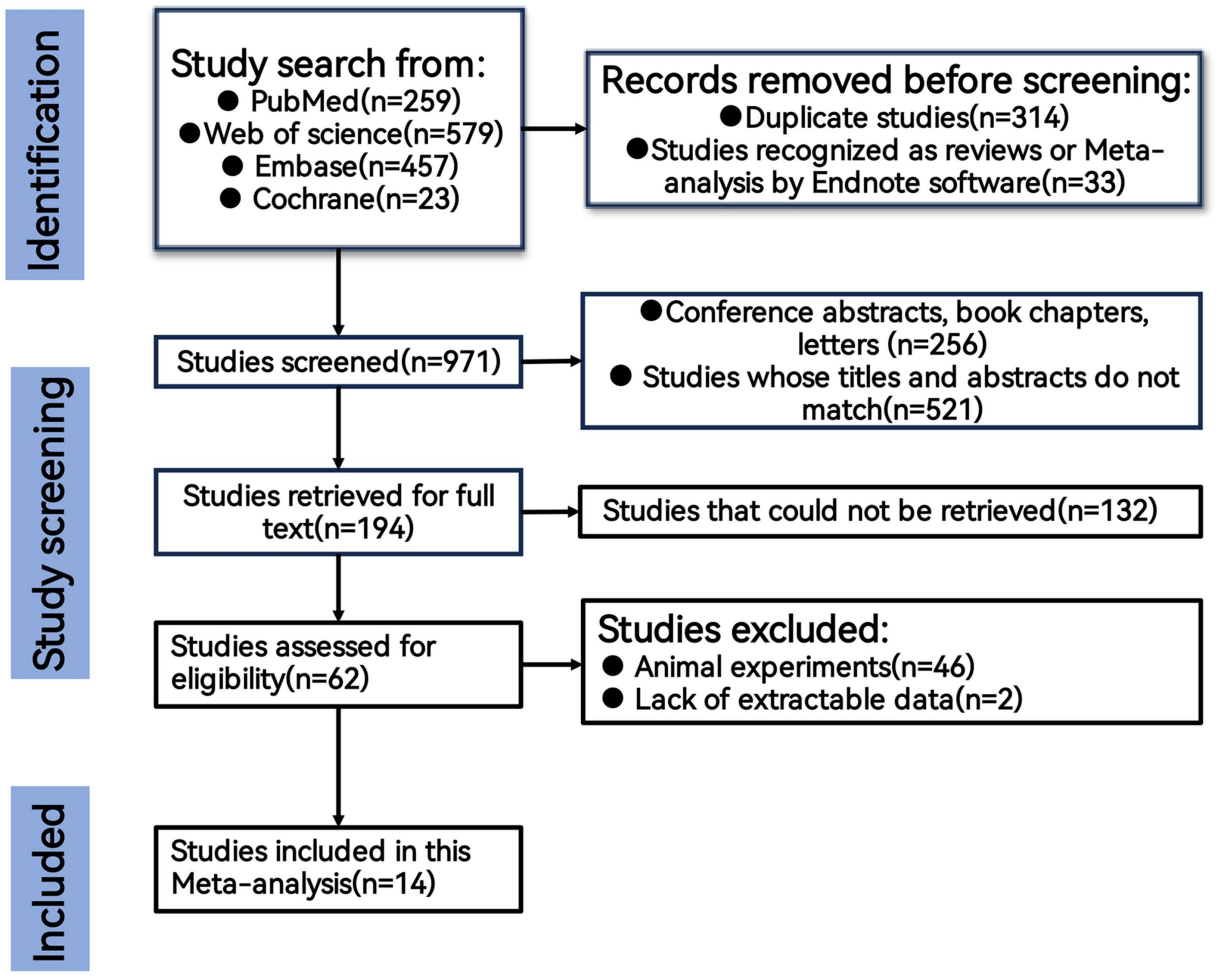
Flow chart of literature screening.

There were 1,428 articles were retrieved from PubMed, Embase, Cochrane, and Web of Science databases. After removing duplicates and Studies recognized as reviews or Meta-analysis by Endnote software, 971 articles remained. Following application of the exclusion criteria, 767 irrelevant articles were eliminated. Subsequently, reviews, case reports, and other irrelevant articles were excluded, resulting in the inclusion of 14 studies comprising 30,752 patients in the meta-tis depicted in Figure 1. The included studies comprised two prospective cohort studies (12, 27), two case–control studies (16, 19), and 10 retrospective cohort studies (13, 17, 18, 20–26). Three articles did not explicitly report the NLR cut-off values (17, 19, 20). All articles were published in English between 2015 and 2024. NLR was measured before ART in three studies (12, 16, 23) and after ART in three studies (17, 19, 20). Mortality was the primary outcome in 10 studies, with two also reporting HRs (95% CI) for recurrence-free survival. The remaining four studies used cardiovascular event rate, lung cancer, hypertension, and new-onset TB as outcome measures. The Newcastle-Ottawa Scale quality assessment indicated that all included studies scored >6, suggesting high quality and low risk of bias (15).

### 3.2 Meta-analysis results

#### 3.2.1 NLR and morality

**Figure 2:**
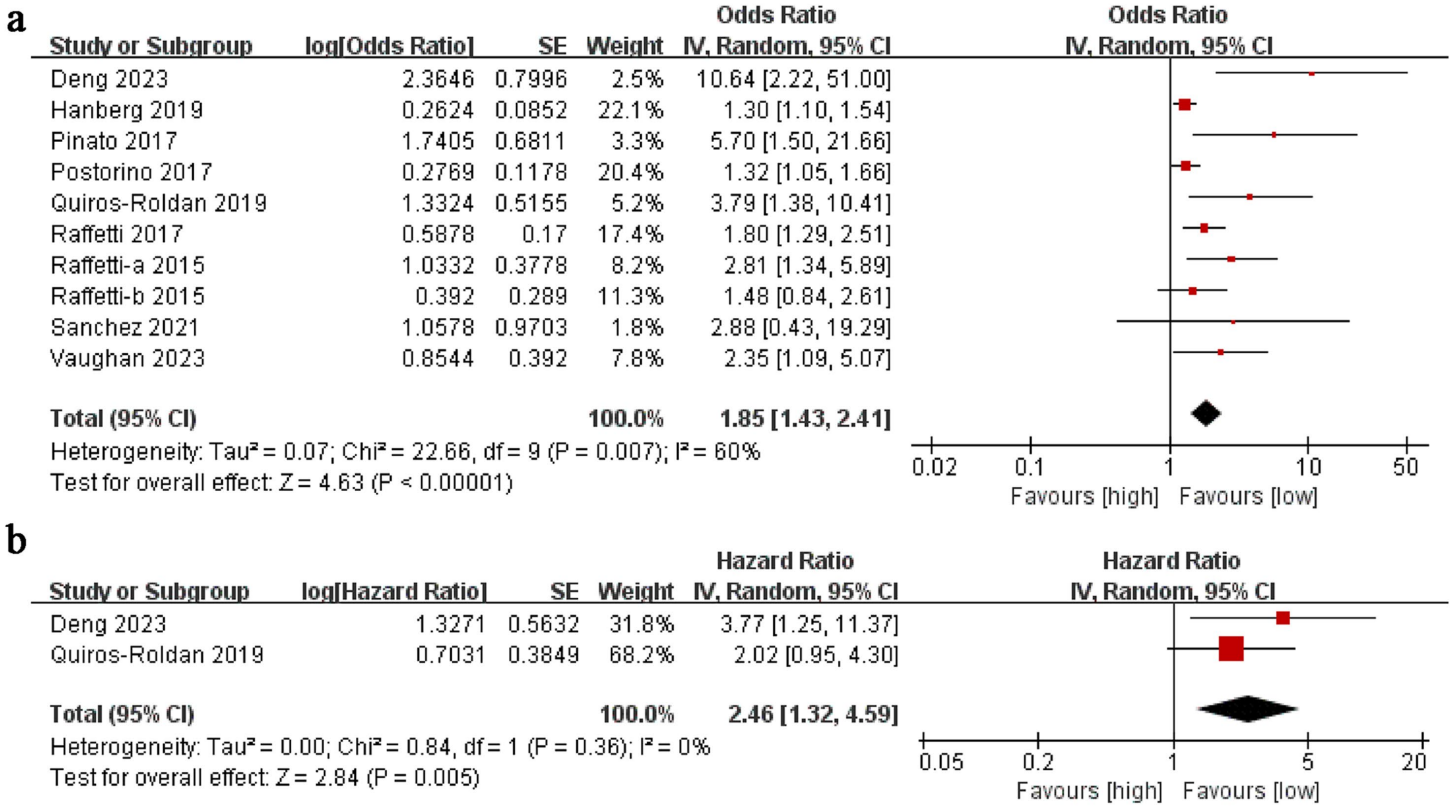
**(A)** Forest plots for the association between NLR and Morality; **(B)** Forest plots for the association between NLR and PFS.

The association between NLR and mortality was investigated. Ten studies involving 25,297 participants were included. A random-effects model analysis of the ORs and 95% CIs indicated that NLR was linked to mortality in HIV-positive patients (OR: 1.85, 95% CI: 1.43–2.41, *p* < 0.00001; Figure 2A), with significant heterogeneity (*I*^2^ = 60%, *p* < 0.00001). To investigate sources of heterogeneity and identify populations where NLR has greater predictive value, subgroup analyses were performed to adjust for confounders. These analyses were based on study type, follow-up duration, timing of testing, sample size, age, NLR cut-off, and whether the patient has a combined lymphoma. The results showed that heterogeneity varied significantly with follow-up duration, timing of testing, sample size, NLR cut-off, and whether the patient has a combined lymphoma. All subgroup predictions were statistically significant (*p* < 0.05), except for the NLR level before ART treatment (Table 2).

**Table 2:** Pooled ORs for mortality in subgroup analyses.

Subgroup	Mortality			
	Study	OR [95%CI]	*p* value	*I* ^2^
Total	10	1.85 [1.43–2.41]	<0.00001	60%
Study design				
Prospective	3	2.08 [1.00–4.35]	0.05	70%
Retrospective	7	1.98 [1.39–2.81]	0.0001	58%
Follow-up				
>3y	5	2.18 [1.38–3.45]	0.0009	75%
≤3y	5	1.73 [1.17–2.56]	0.006	40%
Time of test				
Baseline	2	2.65 [0.91–7.71]	0.07	63%
Post-treatment	2	1.31 [1.14–1.50]	0.0001	0%
Sample size				
≥200	5	1.47 [1.22–1.76]	<0.0001	40%
<200	5	3.57 [2.15–5.91]	<0.00001	0%
Mean/median age				
≥43	4	3.16 [1.46–6.84]	0.004	63%
<43	4	1.77 [1.25–2.49]	0.001	56%
NLR cut-off				
≥4	4	2.88 [1.78–4.64]	<0.0001	0%
<4	4	2.31 [1.33–4.00]	0.003	59%
Lymphomas				
Yes	3	1.79 [1.15–2.79]	0.01	0%
No	7	1.91 [1.39–2.62]	<0.0001	71%

OR, odds ratio; CI, confidence interval; Baseline, NLR was obtained prior to ART treatment; Post-treatment, NLR was obtained after ART treatment; Lymphomas, Whether the patient has a combined lymphoma.

#### 3.2.2 NLR and PFS

The link between NLR and progression-free survival (PFS) in HIV-positive patients was also examined. Two studies comprising 97 participants were included. Pooled analyses of HRs and 95% CIs were conducted using a random-effects model. A high NLR was found to be associated with shorter PFS (HR: 2.46, 95% CI: 1.32–4.59, *p* = 0.005; Figure 2B), mirroring the findings of the mortality study. No significant heterogeneity was detected (*I*^2^ = 0%, *p* = 0.36).

### 3.3 Sensitivity analysis

**Figure 3:**
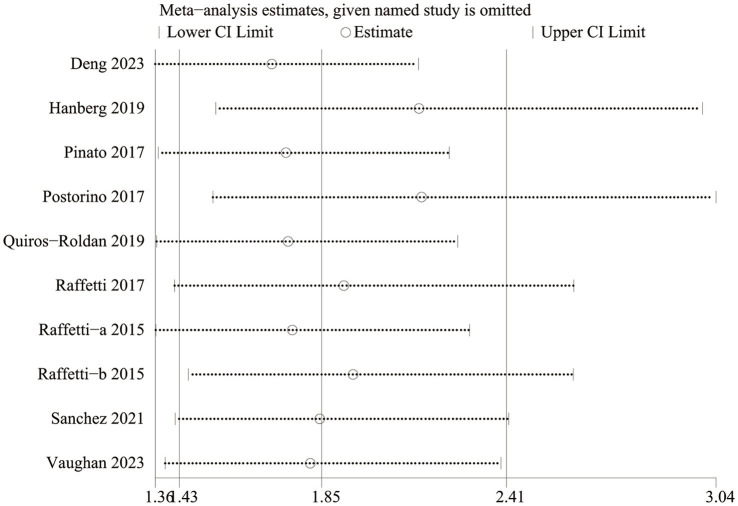
Sensitivity analysis of morality.

Sensitivity analyses for mortality demonstrated that the effect sizes remained stable and within the original range even after sequential exclusion of each study. This indicates that no individual study had a disproportionate impact on the mortality outcome, thereby confirming the robustness and reliability of the analysis (Figure 3).

### 3.4 Publication bias

**Figure 4:**
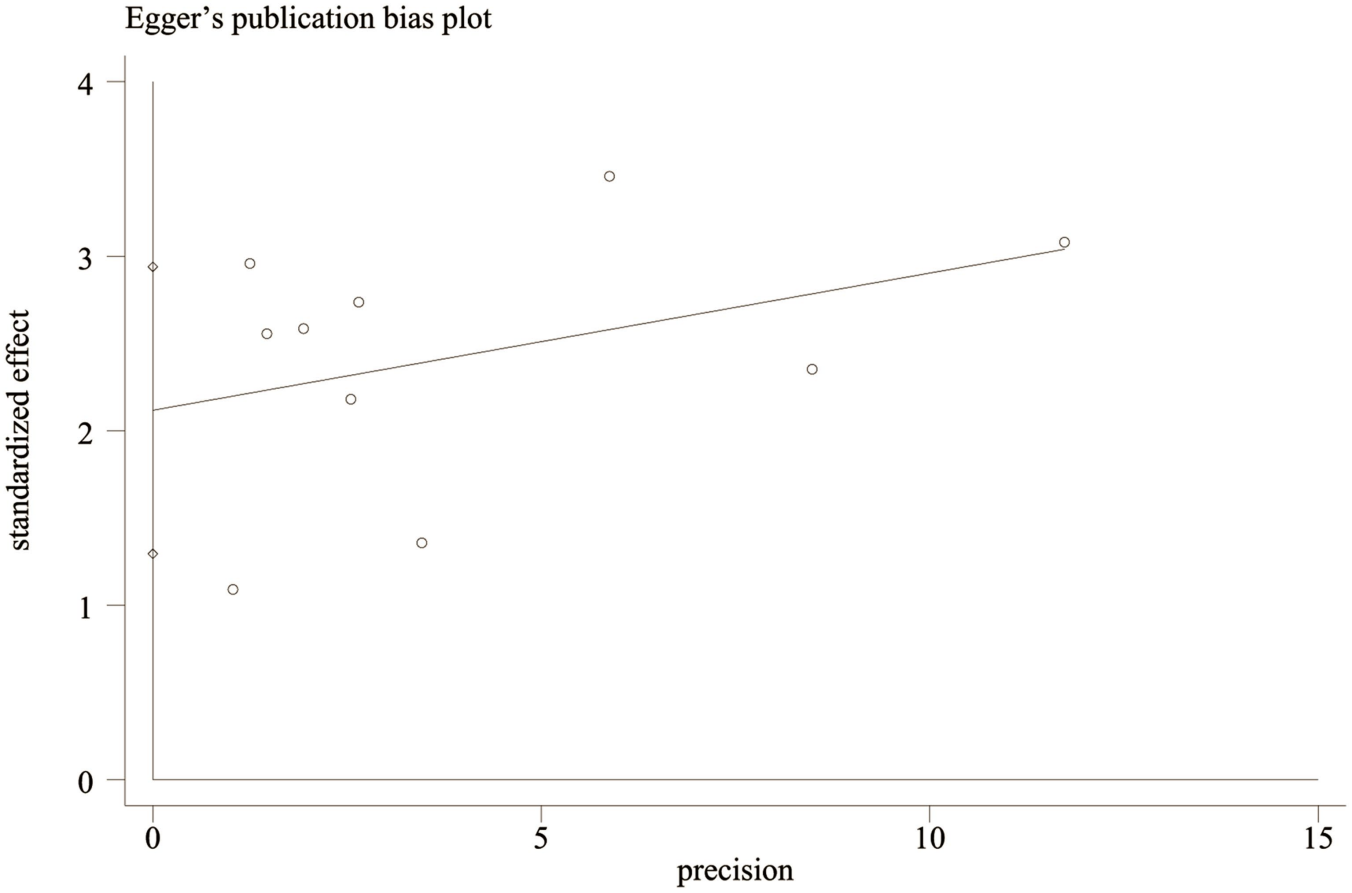
Egger test for publication bias.

**Figure 5:**
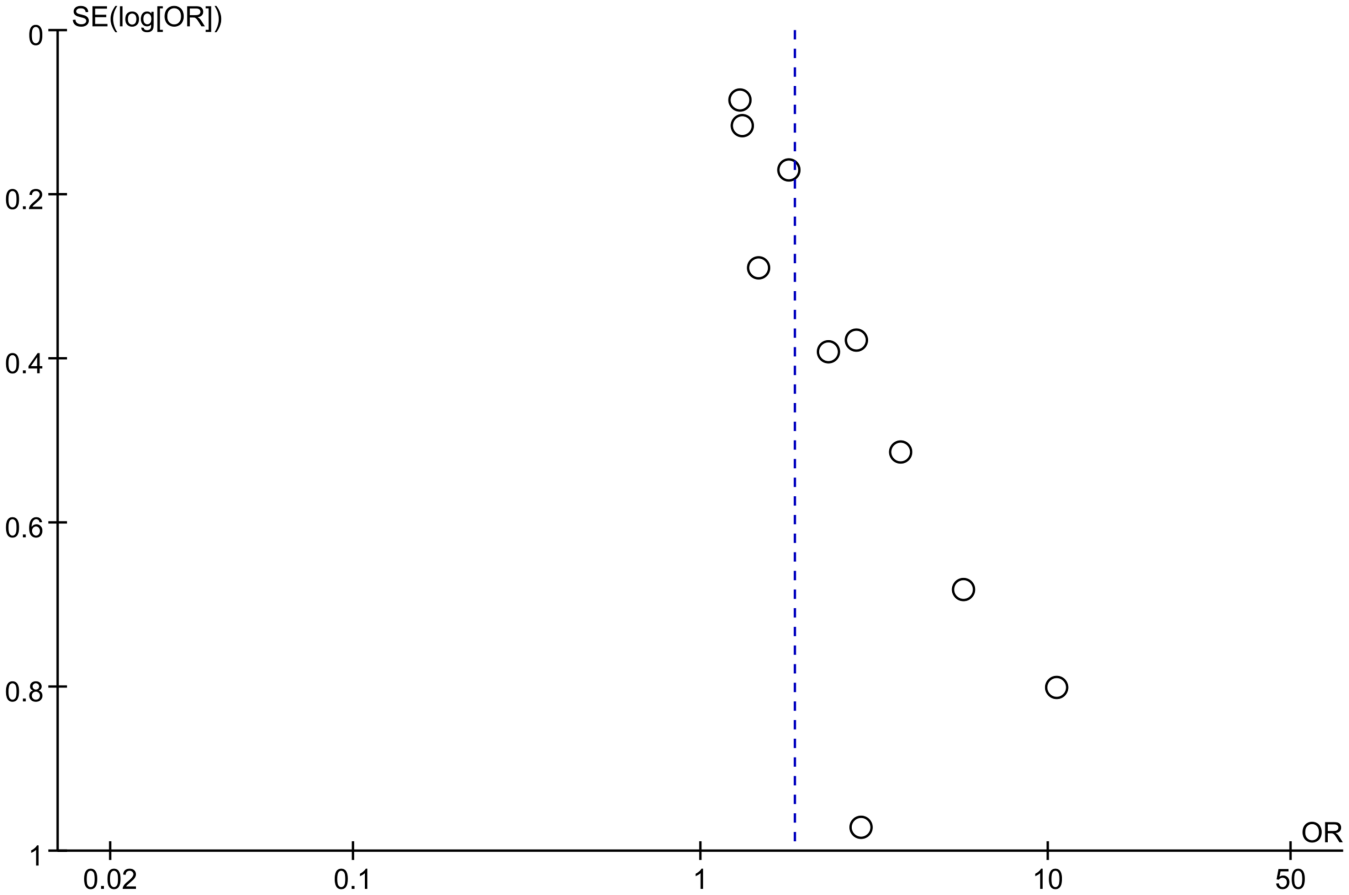
Funnel plot for publication bias.

Egger’s test and funnel plot were used to evaluate the publication bias. The results for the mortality meta-analysis indicated no significant publication bias (Egger’s test *p* < 0.0001; Figures 4, 5). Due to the limited number of studies (*n* = 2), publication bias was not assessed for PFS.

## 4 Discussion

Although the virus transmitted from chimpanzees and monkeys to humans around 100 years ago (28), AIDS has been recognized as an epidemic for over 30 years. Extensive research into the history, pathogenesis, and clinical pathology of AIDS has generated new insights. HIV gradually destroys CD4+ lymphocytes, resulting in lymphocyte depletion and the activation of inflammatory factors in the body (29). Blood-derived parameters offer a simple and reproducible method for evaluating systemic inflammation and can serve as objective biomarkers for predicting patient prognosis (30, 31). Consequently, NLR can be utilized as a monitoring indicator throughout the AIDS cycle to reflect the prognosis of HIV patients.

Here, we executed a comprehensive search of four databases using a predefined search strategy, ultimately including 14 articles with a total of 30,752 patients according to established inclusion and exclusion criteria. Our primary goal was to explore the association between NLR and both mortality and PFS. We utilized Review Manager 5.4 and StataSE 15 to generate forest plots, conduct sensitivity analyses. The findings indicated that a high NLR correlates with a poor prognosis in HIV patients. Due to the limited number of studies (*n* = 2) addressing PFS, sensitivity analyses were not feasible. Nonetheless, preliminary data suggest that elevated NLR is also linked to shorter PFS. Multiple studies have reported findings consistent with ours. For example, Raffetti et al. (25) performed a multicenter cohort study in Italy from 2000 to 2012, utilizing univariate and multivariate analyses with time-independent and time-dependent Cox proportional hazards models to evaluate the relationship between NLR and all-cause mortality in HIV patients. This study involved 8,230 participants (73.1% male) with a mean age of 38.4 years (SD 10.1). Over a median follow-up of 3.9 years, 539 patients died. NLR values exceeding 1.1 were linked to a linearly increased mortality risk (HR 1.80, 95% CI 1.29–2.514).

To investigate the HIV patient subgroups in which NLR has a higher predictive value and to identify sources of heterogeneity, we conducted comprehensive subgroup analyses. The findings revealed that NLR’s predictive value was greater in studies with follow-up durations exceeding 3 years compared to those with shorter follow-ups, aligning with HIV epidemiology. NLR testing performed after ART initiation showed higher predictive value than pre-ART testing. Age-stratified analysis indicated that NLR was more predictive in younger HIV patients than in older ones. Heterogeneity in the results could be attributed to differences in follow-up duration, timing of NLR testing, and sample sizes, and NLR cut-off, and whether the patient has a combined lymphoma (Table 2).

The neutrophil-to-lymphocyte ratio (NLR) reflects the balance between the innate immune system (neutrophils) and the adaptive immune response (lymphocytes), both critical components of the body’s immune system. Lymphocytes, which are the primary target cells of HIV, play a significant role in determining this ratio. A high NLR indicates an increase in neutrophils and/or a decrease in lymphocytes. Elevated neutrophil levels often indicate heightened inflammation in the body. Inflammation serves as a protective mechanism against infections and injuries, activating both innate and adaptive immune responses to combat pathogens and promote tissue repair. In some cases, chronic inflammation may persist throughout the lifespan (32). HIV infection is characterized by the expression of pro-inflammatory cytokines that regulate HIV replication and T-cell apoptosis throughout the virus’s life cycle (33). Furthermore, a cytokine-rich environment may allow tumor cells to evade immune surveillance, resulting in adverse clinical outcomes (34–36). Previous research has linked neutrophils to the development of various diseases. For instance, prolonged neutrophil elevation can cause endothelial dysfunction and contribute to hypertension by activating inflammatory pathways and inducing oxidative stress (37). Moreover, neutrophils migrate to the vessel wall, producing proinflammatory and atherogenic effects, and their recruitment is linked to plaque rupture (38). HIV patients are known to have a high incidence of Pneumocystis carinii pneumonia and various tumors, indicating an impaired immune system. HIV primarily targets CD4 T cells, and the CD4 count is considered the most critical predictor of clinical progression in HIV patients. Decreased CD4 T cell counts may impair immunosurveillance and cytotoxicity against cancer cells. Additionally, low lymphocyte counts have been associated with a poorer prognosis in patients with coronary artery disease and unstable angina, although the underlying mechanism remains unclear. Moreover, T-lymphocytes play a crucial role in the immune response against tuberculosis infections. While our study provides significant insights into the prognostic value of NLR in AIDS patients, it is crucial to consider other relevant research in the field. A recent study by Ron et al. examined the CD4/CD8 ratio and CD8+ T-cell count as predictors of non-AIDS mortality in individuals living with HIV. Through a systematic review and meta-analysis, they found that a low CD4/CD8 ratio, particularly values below 0.5, was associated with increased non-AIDS and all-cause mortality risk (OR 3.64; 95% CI 3.04–4.35; I2 = 0.00%). While the meta-analysis of CD8+ T-cell counts was hindered by methodological variations, the systematic review suggested a potential negative prognostic impact of higher values in the long term. Similar to our research on NLR, these findings underscore the importance of immune markers in predicting HIV prognosis. However, our study focused on a different aspect of the immune response, specifically the neutrophil-to-lymphocyte ratio (39). In summary, NLR reflects the interaction between HIV and the immune system, representing the balance between innate (neutrophil) and adaptive (lymphocyte) immune responses.

Our meta-analysis has several limitations. Primarily, this meta-analysis with few prospective studies, potentially introducing confounding factors that may have affected the reliability of our results. Secondly, the heterogeneity in NLR cutoff values across the included studies, ranging from 1.1 to 6, may have introduced inherent variability in our meta-analysis due to inconsistent data. We recommend that future researchers establish a standardized cutoff value to enhance comparability across studies. Thirdly, the limited number of studies (*n* = 2) examining PFS among the included studies prevented us from conducting sensitivity analyses and Egger’s test, potentially biasing the results. Finally, some of the included studies also explored cardiovascular event rates, tuberculosis positivity, and lung cancer incidence in HIV patients. While our analysis indicated a positive association between elevated NLR and these adverse clinical outcomes, further investigation was constrained by the scarcity of studies for each indicator. Our study also has several strengths. Firstly, this is the inaugural meta-analysis examining the link between NLR and prognosis in HIV-positive patients. Secondly, we encompassed a substantial patient cohort, bolstering the robustness of our findings. Thirdly, we performed comprehensive subgroup analyses to pinpoint the populations in which NLR holds heightened predictive value. Lastly, sensitivity analyses underscored the stability and reliability of our conclusions.

## 5 Conclusion

NLR exhibits notable prognostic value in individuals with HIV. Higher NLR levels are linked to increased mortality and shorter PFS among HIV-positive patients. This study’s results endorse the creation of a prognostic model that includes immunoinflammatory markers like NLR to better predict outcomes for these patients. However, due to the limitations of this study, such as the predominance of retrospective studies, significant heterogeneity, and a small sample size, there is a need for future prospective, multicenter studies with larger cohorts and longer follow-up periods to confirm these findings.

## Data Availability

The original contributions presented in the study are included in the article/Supplementary material, further inquiries can be directed to the corresponding author/s.
