# Supplementary material for: Predictive value of neutrophil to lymphocyte ratio for the clinical outcomes of acquired immune deficiency syndrome: a systematic review and meta-analysis
Source: Front Med (Lausanne). 2025 Feb 5;12:1503614. doi: 10.3389/fmed.2025.1503614 (PMC11835827; doi:10.3389/fmed.2025.1503614)
Supplement: Supplementary file 1 [file Table_2.DOCX]

**Supplementary materials**

**Supplementary Table 1 Detailed Search Format**

1. Pubmed---259

(((("Neutrophils"[Mesh]) OR (((((((((Neutrophil) OR (Polymorphonuclear Leukocyte)) OR (Polymorphonuclear Leukocytes)) OR (Polymorphonuclear Neutrophils)) OR (Polymorphonuclear Neutrophil)) OR (LE Cells)) OR (LE Cell)) OR (Neutrophil Band Cells)) OR (Neutrophil Band Cell))) AND (("Lymphocytes"[Mesh]) OR (((((Lymphocyte) OR (Lymphoid Cells)) OR (Cell, Lymphoid)) OR (Cells, Lymphoid)) OR (Lymphoid Cell)))) AND (Ratio)) AND (("Acquired Immunodeficiency Syndrome"[Mesh]) OR (((((((Acquired Immune Deficiency Syndrome) OR (Acquired Immuno-Deficiency Syndrome)) OR (Acquired Immuno Deficiency Syndrome)) OR (Acquired Immuno-Deficiency Syndromes)) OR (Acquired Immunodeficiency Syndromes)) OR (AIDS)) OR (HIV)))

1. Embase-457


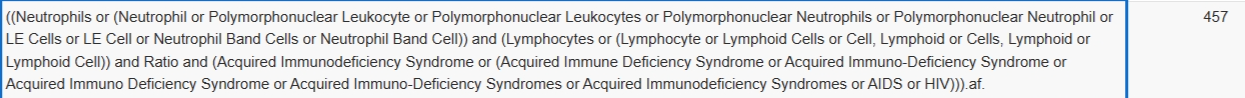


1. Cochrane---23


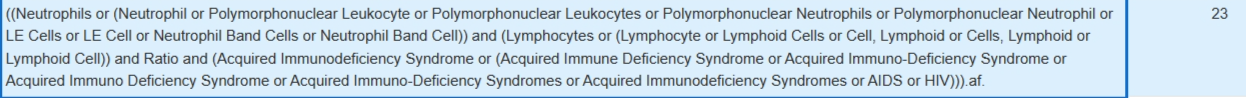


1. Web of science--579

((((Neutrophils) OR (((((((((Neutrophil) OR (Polymorphonuclear Leukocyte)) OR (Polymorphonuclear Leukocytes)) OR (Polymorphonuclear Neutrophils)) OR (Polymorphonuclear Neutrophil)) OR (LE Cells)) OR (LE Cell)) OR (Neutrophil Band Cells)) OR (Neutrophil Band Cell))) AND ((Lymphocytes) OR (((((Lymphocyte) OR (Lymphoid Cells)) OR (Cell, Lymphoid)) OR (Cells, Lymphoid)) OR (Lymphoid Cell)))) AND (Ratio)) AND ((Acquired Immunodeficiency Syndrome) OR (((((((Acquired Immune Deficiency Syndrome) OR (Acquired Immuno-Deficiency Syndrome)) OR (Acquired Immuno Deficiency Syndrome)) OR (Acquired Immuno-Deficiency Syndromes)) OR (Acquired Immunodeficiency Syndromes)) OR (AIDS)) OR (HIV))) (Topic) and Preprint Citation Index (Exclude – Database)

**Supplementary Table 2 Quality assessment of the included literature**

(A). Evaluation of the quality of cohort studies

| Study | Selection | | | | Comparability | | Outcome | | |
| --- | --- | --- | --- | --- | --- | --- | --- | --- | --- |
|  | Representative-ness | Selection of  non-exposed | Ascertainment  of exposure | Outcome not present at start | Comparability on most important factors | Comparability on other risk factors | Assessment of outcome | Long enough follow-up (median≥1 year) | Adequacy  (completeness) of follow-up |
| Pinato et al. | * | * | * | * | - | - | * | * | - |
| Raffetti-a et al. | * | * | * | * | - | - | * | * | - |
| Raffetti-b et al. | * | * | * | * | - | - | * | * | * |
| Postorino et al. | * | * | * | * | - | - | * | * | - |
| Raffetti et al. | * | * | * | * | - | - | * | * | * |
| Quiros-Roldan et al. | * | * | * | * | - | - | * | * | * |
| Vaughan et al. | * | * | * | * | - | - | * | * | * |
| Miyahara et al. | * | * | * | - | - | - | * | * | - |
| Hanberg et al. | * | * | * | * | - | - | * | * | - |
| Quiros-Roldan et al. | * | * | * | * | - | - | * | * | * |
| Deng et al. | * | * | * | * | * | - | * | * | - |
| Sanchez et al. | * | * | * | * | * | - | * | * | - |

*indicates criterion met; - indicates significant of criterion not met.

(B). Case-control quality assessment

| Study | Selection | | | | Comparability | | Outcome | | |
| --- | --- | --- | --- | --- | --- | --- | --- | --- | --- |
|  | Appropriateness of case ascertainment | Representativeness of cases | Selection of controls | Ascertainment of controls | Comparability on most important factors | Comparability on other risk factors | Ascertainment of exposure factors | Identical methods were used to determine exposure factors | non-response rate |
| Baluku et al. | * | * | - | * | - | - | * | * | * |
| Ou-Yang et al. | * | * | * | * | - | - | * | * | * |

*indicates criterion met; - indicates significant of criterion not met.
